# Long-Term Outcome of Pediatric Patients with Anti-NMDA Receptor Encephalitis in a Single Center

**DOI:** 10.3390/children10020182

**Published:** 2023-01-18

**Authors:** Pei-Yu Wu, Ching-Shiang Chi, Chi-Ren Tsai, Yao-Lun Yang, Hsiu-Fen Lee

**Affiliations:** 1 Division of Pediatric Neurology, Children’s Medical Center, Taichung Veterans General Hospital, 1650, Taiwan Boulevard Sec. 4, Taichung 407, Taiwan; 2 Department of Post-Baccalaureate Medicine, College of Medicine, National Chung Hsing University, 250, Kuo Kuang Rd., Taichung 402, Taiwan

**Keywords:** anti-N-methyl-D-aspartate receptor (NMDA) encephalitis, children, mRS, outcome, treatment

## Abstract

Background: Anti-N-methyl-D-aspartate (NMDA) receptor encephalitis is the most common autoimmune encephalitis in children. There is a high probability of recovery if treated promptly. We aimed to analyze the clinical features and long-term outcomes of pediatric patients with anti-NMDA receptor encephalitis. Method: We conducted a retrospective study with definite diagnoses of anti-NMDA receptor encephalitis in 11 children treated in a tertiary referral center between March 2012 and March 2022. Clinical features, ancillary tests, treatment, and outcomes were reviewed. Results: The median age at disease onset was 7.9 years. There were eight females (72.7%) and three males (27.3%). Three (27.3%) patients initially presented with focal and/or generalized seizures and eight (72.7%) with behavioral change. Seven patients (63.6%) revealed normal brain MRI scans. Seven (63.6%) had abnormal EEG results. Ten patients (90.1%) received intravenous immunoglobulin, corticosteroid, and/or plasmapheresis. After a median follow-up duration of 3.5 years, one patient was lost to follow-up at the acute stage, nine (90%) had an mRS ≤ 2, and only one had an mRS of 3. Conclusions: With the early recognition of anti-NMDA receptor encephalitis based on its clinical features and ancillary tests, we were able to treat patients promptly with first-line treatment and achieve favorable neurological outcomes.

## 1. Introduction

The N-methyl-D-aspartate (NMDA) receptor is an ionotropic and excitatory glutamate receptor, which plays important roles in memory, learning and cognitive functions. Each receptor is a heteromeric complex, consisting of two N1 subunits and either two N2 or N3 subunits [1]. When antibodies directly bind to the N1 subunit of the NMDA receptor, there is a reduction in the surface density of the NMDA receptor due to receptor internalization, which leads to NMDA receptor hypofunction [2].

Anti-NMDA receptor encephalitis was first proposed as a new immune-mediated encephalitis in young adults in 2008 [3]. It is also increasingly recognized in pediatric populations and it is the most common autoimmune encephalitis in children.

There are four phases during the disease course. The prodromal phase is characterized by fever, respiratory tract infection, nausea, and vomiting. Second, the patients at the psychiatric phase present with behavioral change, irritability and sleep dysfunction, which may last for 1–2 weeks. Third, the neurological phase is characterized by seizures and movement disorder. Without appropriate treatment, some cases gradually progress to autonomic instability, reduced consciousness, or hypoventilation. This is followed by the recovery phase and the slowest to recover are the cognitive and psychiatric functions [4]. Clinical outcomes depend on the etiology of the condition; the presence of a tumor is less common in children compared to adolescents and adults [4]. Other possible etiologies, including post-infection with mycoplasma and herpes simplex encephalitis, have been reported [4,5]. Shu et al. showed an association with the HLA class II allele DRB1*16:02 [6]. Early recognition of the disease could lead to timely treatment and achieve better neurological outcomes [7,8].

First-line treatment of this disease includes intravenous immunoglobulin, corticosteroid, and plasmapheresis; second-line treatment includes rituximab or cyclophosphamide, or both [9,10]. However, children may be more vulnerable to chemotherapy than adults, as there could be adverse events in younger patients, including cognitive impairment and secondary tumors. As shown in the literature, 79.2% (19/24) and 85.1% (149/175) of pediatric patients with anti-NMDA receptor encephalitis who received first-line treatment with or without second-line treatment had an mRS ≤ 2 after 12 months from disease onset [11,12].

The aim of this study is to analyze the clinical features and long-term outcomes of the patients who had a definite diagnosis of anti-NMDA receptor encephalitis after receiving any first-line treatment at a single tertiary referral center over a 10-year period.

## 2. Materials and Methods

This is a retrospective study. From March 2012 to March 2022, patients aged less than 18 years who were diagnosed with anti-NMDA receptor encephalitis at a single tertiary referral center were enrolled.

All patients met the criteria of a definite diagnosis of anti-NMDA receptor encephalitis, which was based on the detection of anti-NMDA receptor antibodies in the cerebrospinal fluid (CSF) and relevant clinical manifestations, such as psychiatric symptoms, seizures, dystonia, decreased level of consciousness, and autonomic dysfunction [13]. The antibodies were sent to a laboratory at the Uni Pharma company in Taiwan. Patients with other diagnoses, such as psychiatric problems, viral encephalitis, post-infectious encephalitis, pediatric autoimmune neuropsychiatric disorders associated with streptococcal infections (PANDAS), inborn errors of metabolism (including urea cycle disorders) and toxin, or drug overdoses, were excluded.

All patients received complete history taking, neurological examination, laboratory workups (including spinal tapping), electroencephalograms (EEG), and brain magnetic resonance imaging (MRI). Abdominal imaging studies were arranged for potential tumor screening after anti-NMDA receptor encephalitis was diagnosed. They all received first-line treatment at the acute stage, including methylprednisolone pulse therapy (MTP) (30 mg/kg (max. 1000 mg)) for 5 days, IVIG (400 mg/kg) for 5 days, and/or plasmapheresis every other day for a 5-day course. When the patient presented with a sleep disorder or behavior problem, symptomatic medications were prescribed.

During the follow-up period, abdominal imaging studies were conducted every 6 months. Follow-up EEG and brain MRI were performed at least 6 to 12 months after the diagnosis. As for further treatment, if patients still had seizures, anti-seizure medications (ASMs) were prescribed. Some patients received low-dose IVIg (0.5 g/kg/day) monthly if they had a poor response to ASMs after the acute stage [14]. For the assessment of clinical outcome, we used the Anti-NMDA Receptor Encephalitis One-Year Functional Status (NEOS) score, modified Rankin Scale (mRS) and Clinical Assessment Scale in Autoimmune Encephalitis (CASE) score. The NEOS score has five characteristics, including intensive care unit (ICU) admission, treatment initiation within 4 weeks of symptom onset, lack of improvement at 4 weeks from treatment, abnormal MRI, and CSF white cell count of >20 cells/µL, to evaluate initial neurological status. There is one point for each characteristic with a maximum sum score of 5. A higher NEOS score, especially 4 or 5 points, was associated with a lower probability of 1-year functional status [15]. The mRS and CASE score were also assessed at disease onset, 6 months after disease onset, 1 year after disease onset and the last visit. mRS is used to measure the degree of disability or dependence in the daily activities of a patient who had a stroke or other neurological disorder. It ranges from 0 to 6, as follows: 0, no symptoms; 1, no significant disability despite symptoms; 2, slight disability and being unable to carry out previous activities, but being able to walk without assistance; 3, moderate disability and being unable to attend to own bodily needs without assistance, but still being able to walk; 4, severe moderate disability and need help in both walking and own bodily needs; 5, severe disability with bedridden status; 6, death [16]. The CASE score has nine key symptoms, including seizure, memory dysfunction, psychiatric symptoms, consciousness, language problems, dyskinesia/dystonia, gait instability/ataxia, brainstem dysfunction and weakness. Each symptom has 0 to 3 points according to its severity. The CASE score is a sum of the score from nine key symptoms, with a maximum score of 27. A higher score indicates a worse condition [17].

The demographic data, clinical features, laboratory data, EEG features, neuroimaging findings, duration between symptom onset and treatment, treatment strategies, and duration of the first hospitalization were analyzed. This study was approved by the Institutional Review Board of Taichung Veterans General Hospital (TCVGH IRB CE20030A).

## 3. Results

### 3.1. Clinical Data

Demographic data, clinical manifestations, ancillary tests, and treatments of the 11 patients are shown in Table 1 and Table 2. There were three males and eight females included in this study. The median age at disease onset was 7.9 years, ranging from 2.5 to 17.9 years old. Five patients (45.5%) had febrile illness as the prodromal symptoms. At the beginning of the disease course, eight patients (72.7%) presented with behavioral change, such as irritable mood, aggressiveness, or exaggerated talk, while the other three (27.3%) presented with seizures. Individual 1 had focal seizures that evolved to generalized tonic–clonic seizures; individual 2 had focal seizures; and individual 9 initially exhibited three episodes of generalized tonic–clonic seizures, followed by clusters of focal seizures. During the disease course, all patients (100%) had behavioral changes, including mood, and/or personality changes. Seven patients (63.6%) had orofacial dyskinesia or speechlessness, and six patients (54.5%) had seizures or autonomic dysfunction at the acute stage. In addition, some of the patients demonstrated a complex disease course. Nine out of eleven patients had symptoms and signs of neurological change; and six out of nine patients exhibited autonomic dysfunction, including apnea, desaturation, tachycardia, or bradycardia. These patients needed intensive care in terms of frequent monitoring of vital signs and/or intubation.

Four patients had CSF pleocytosis and one patient had elevated CSF protein levels. No patients had evidence of previous herpes simplex virus encephalitis. The EEG at disease onset revealed that 63.6% (7/11) of individuals had abnormal results, including background slowing, absence of sleep spindles, or continuous polymorphic slowing at the left or right hemisphere. Seven out of eleven patients (63.6%) initially had a normal brain MRI scan; while four (36.4%) had abnormal findings, including signal changes over the hippocampi, thalamus, putamen, frontal, temporal, or parietal lobes. Only one patient had a tumor and the pathological finding revealed a right adrenal cortical adenoma.

### 3.2. Treatment

The median duration between disease onset and initiation of treatment was 21 days, ranging from 7 to 90 days. All patients received MTP pulse therapy. Ten out of eleven patients (90.9%) received administration of IVIG and four (36.4%) received plasmapheresis. One patient who had a tumor underwent surgical excision. In addition, six out of ten patients (60%) with seizures received ASMs at the acute stage of the disease. Individuals 1, 3, and 8 received chloral hydrate, due to agitation and/or poor sleep. Individuals 9, 10, and 11 needed estazolam or lorazepam because of a sleep disorder. Individuals 10 and 11 received aripiprazole or haloperidol for behavior problems.

### 3.3. Follow-up

One patient was lost to follow-up at the acute stage of anti-NMDA receptor encephalitis. The median duration of the follow-up period was 3.5 years, ranging from 1 to 6 years. Among these 10 patients, only individual 8 experienced recurrent anti-NMDA receptor encephalitis and presented with strange behavior 4 months after the first episode. Three patients, individuals 1, 2, and 9, had persistent seizures 3 months after disease onset. Individual 1 received low-dose IVIG monthly 13 times and she was free from seizures after the last dose of IVIG. Individual 2 had previously following a ketogenic diet and received ACTH therapy. She also had received multiple ASMs, such as topiramate, valproate, vigabatrin, and clobazam, due to uncontrolled seizures. However, minimal clinical efficacy was noted. After prescribing low-dose IVIG monthly for 31 courses, focal seizures were still noted, but with decreased frequency and improvement in language and cognitive functions. Individual 9 received low-dose IVIG monthly 17 times for seizure control. Later, he was prescribed oxcarbazepine and clobazam, and was free from seizures for more than 1 year. Except for one patient (individual 2) who had focal seizures, all of the patients were free from seizures at the last follow-up. Aside from ASMs, all patients did not receive other symptomatic medications for sleep disorder or behavior problems after discharge.

**Figure 1:**
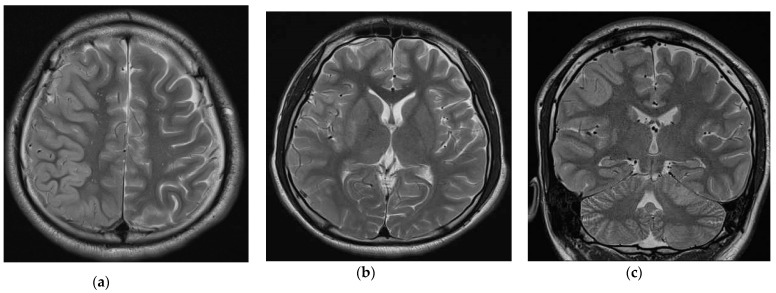
Brain MRI for individual 9 at day 15 of disease onset. (**a**,**b**) The axial brain MRI (TE 103 ms; TR 3820 ms) shows diffuse cortical swelling over the right frontal and temporal areas and T2WI high signal intensity over the right putamen and right thalamus. (**c**) The coronal MRI (TE 83 ms; TR 4000 ms) shows T2WI high signal intensity over the right putamen, right thalamus, and left cerebellum.

Eight patients had a follow-up EEG 6 to 12 months after disease onset; five showed abnormal results, including background slowing, continuous polymorphic slowing, and focal spikes. Four patients had a follow-up brain MRI scan at 6 to 12 months after the disease onset; two had generalized brain atrophy and relatively small hippocampi, one patient demonstrated signal changes over the bilateral frontal and parietal regions, as in the initial image study, and one showed significant improvement of signal change over the right thalamus, putamen, frontal, and temporal regions (Figure 1). At disease onset, the median NEOS score was 2 points, ranging from 1 to 4 points. The percentage of patients with ≤2 points on the mRS scale at the last visit was 90% (9/10). The CASE scores at the last visit ranged from 0 to 4, largely comprising memory dysfunction, language problems and/or controlled seizures. Serial clinical outcomes evaluated via mRS and the CASE score are shown in Table 3. The median mRS of the patients aged ≥12 years at disease onset, 6 months, and 12 months was 3 points (range, 2 to 4), 1 point (range, 0 to 1) and 0.5 points (range, 0 to 1), respectively, and for the patients aged <12 years at disease onset, 6 months and 12 months, the median mRS was 3 points (range, 2 to 3), 1 point (range, 0 to 4), and 1 point (range, 0 to 3). The median CASE scores of the patients aged ≥12 years at disease onset, 6 months and 12 months were 7 points (range, 3 to 8), 1.5 points (range, 0 to 3), and 1 point (range, 0 to 2), respectively, while for the patients aged <12 years at disease onset, 6 months and 12 months, the scores were 6 points (range, 2 to 7), 1 point (range, 0 to 7), and 1 point (range, 0 to 5), respectively.

## 4. Discussion

The main findings of our study were that most of the pediatric patients who were treated with first-line therapy had favorable neurological outcomes; and most patients did not need long-term ASM use after the acute stage.

The clinical manifestations and disease course of pediatric anti-NMDA receptor encephalitis have been comprehensively described in the literature. The results showed some differences compared with adult patients. A smaller proportion of pediatric patients had tumors compared with adults, which was consistent with our report that only one patient had a tumor [8,11]. In addition, our study showed female predominance, as in previous results [8,18,19]. Neurological symptoms, such as seizures and dyskinesia, were more common than psychiatric symptoms as the initial signs of presentation in children in most of the previous cohorts [7,20]. However, our study showed a higher percentage of patients with behavioral changes, including mood, and/or personality changes (72.7%), as the initial signs of presentation. The remaining three patients without initial behavioral changes had focal seizures in clusters, with or without evolving to bilateral tonic–clonic seizures, which was consistent with a Chinese study that reported focal seizures were the most common seizure type in anti-NMDA receptor encephalitis patients [21].

Although EEG and brain MRI are not diagnostic criteria of anti-NMDA receptor encephalitis, both not only serve as adjunctive diagnostic modalities, but also provide some prognostic information [22,23,24,25,26]. In our study, seven individuals presented with abnormal EEG findings initially. Of these seven patients, four patients demonstrated background slowing, which was the most common finding, and this was consistent with previous studies [22,23,24]. In addition, another unique EEG pattern frequently observed in adult patients is extreme delta brush [27]. However, our patients did not have this EEG pattern. Regarding brain MRI, there were no specific patterns. Abnormal MRI findings were not correlated with the prognosis of the condition, while hippocampal lesions might be risk factors for a poor prognosis in anti-NMDA receptor encephalitis patients, as shown in individual 2 [25,26,28,29,30]. These non-specific features provide pediatric neurologists with some useful information for the diagnosis of patients with seizures and psychiatric problems who were previously healthy children.

We observed that 90% of the individuals had an mRS ≤ 2 and achieved favorable neurological outcomes, even though our patients showed complex disease courses. Most of the patients were responsive to first-line therapy, except for one patient, who had an mRS of 3 points at the last follow-up. This patient had a younger age at disease onset, was further from disease onset, and demonstrated milder severity, as indicated by the CASE score. The results were similar to those from the other two Asian cohorts, in which 77% and 83.3% of patients had a low mRS after first-line therapy [20,31]. Sakpichaisakul et al. also reported that first-line therapy, especially IVIG, was the most important treatment method for improving neurological outcomes 6 to 12 months after disease onset [32]. IVIG administration in individuals with anti-NMDA receptor encephalitis would be capable of achieving a favorable outcome, and second-line regimens, such as chemotherapy, could be avoided to decrease the possibility of impaired cognitive function or secondary tumors.

As for different outcomes based on age, Shim et al. showed a trend with a slower rate of recovery according to the CASE scores in a younger onset group (aged <12 years), although the data did not show any statistically significant differences [11]. Our study showed that individuals 1 and 2 aged <12 years displayed a slower rate in recovery via the CASE score, compared with others at 6 months after disease onset. In addition, Yeshokumar et al. mentioned that a younger age at disease onset (<12 years of age) was not associated with neurological disability at the long-term follow-up; however, they reported that it may be associated with worse long-term adaptive behavior [33]. In the present study, a similar median mRS was recorded in the evaluation of neurological disability at the last follow-up between those aged <12 years of age (median mRS: 0 point) and aged ≥12 years of age (median mRS: 0.5 point). Therefore, age at disease onset might be an important factor associated with the recovery rate and long-term behavior problems of these patients. In addition to the risk of behavior problems, clinicians should also be advised to monitor patients for difficulties with attention and learning, especially as children continue to develop.

Six out of ten patients (60%) with seizures received ASMs at the acute stage. Three patients had persistent seizures after the acute stage; however, only one patient, individual 2, still had focal seizures at the last follow-up. She received a low-dose monthly immunoglobulin injection. As in previous studies, long-term use of ASMs may not be necessary in both pediatric and adult patients [20,21,28]. Most pediatric patients present with acute symptomatic seizures due to complement activation, antigenic modulation, or recruitment of immune cells. Therefore, most cases demonstrated a good response to immunotherapy and a good prognosis, with mild or no neurological sequelae, and without persistent seizures [21]. Patients with autoimmune encephalitis caused by intracellular antibodies had a higher risk of autoimmune-associated epilepsy than those with surface antibodies [34]. However, we still should consider autoimmune-associated epilepsy for patients with intractable seizures after anti-NMDA receptor encephalitis. According to previous studies, ASMs may be ineffective and may provide an incomplete response to epilepsy surgery [35]. Based on our observations, we speculate that low-dose immunoglobulin injections might be a feasible therapy in such individuals.

There were some limitations in our study. First, this was a retrospective study, rather than a randomized controlled study. Second, there was a small sample size and evidence of heterogeneity in the patients and follow-up duration. Further research is needed to confirm our findings.

## 5. Conclusions

Patients with anti-NMDA receptor encephalitis achieved favorable neurological outcomes after receiving first-line therapy. EEG and brain MRI provided useful additional information for the diagnosis of these patients. Patients who had persistent seizures after the acute stage of the disease exhibited a good response to low-dose IVIg administration. Further investigation in terms of a large sample size that would reduce the heterogeneity of the patient population is needed.

## Figures and Tables

**Table 1:** Clinical manifestations, ancillary tests, and treatment of 11 patients with anti-NMDA receptor encephalitis.

Pt	Sex	Age at Disease Onset (Years)	Prodromal s/s				Initial s/s	Overall Clinical Manifestations								CSF Analysis				Tumor	EEG	Brain MRI	Hosp Duration (Days)	Time to Treatment (Days)	Treatment
			Fever	U	G	H		BC	S	O	Sz	A	D	Hal	C	WBC (/cumm)	Protein (15–45 mg/dL)	OCB	IgG Index (0.34–0.58)						MP/IVIG/PP
1	F	7.1	N	N	N	N	Sz	Y	Y	N	Y	Y	Y	Y	Y	0	25	NA	NA	N	CPS, Lt	Normal	26	50	Y/Y/N
2	F	3.2	N	N	N	N	Sz	Y	Y	Y	Y	Y	Y	N	Y	48	22	NA	NA	N	BS/CPS, Lt	Abnormal (Lt hippocampus)	56	47	Y/Y/N
3	F	2.5	Y	Y	Y	N	BC	Y	N	Y	N	N	Y	N	Y	0	21.3	Neg	0.653	N	BS/AS	Normal	73	14	Y/Y/Y
4	F	13.8	N	N	Y	N	BC	Y	N	N	N	N	N	N	N	33	20.5	NA	NA	Y	Normal	Normal	NA*	20	Y/N/N
5	M	15.5	N	Y	Y	Y	BC	Y	Y	Y	Y	N	N	N	N	11	25.9	Neg	0.289	N	BS	Abnormal (Lt frontal region)	5 **	21	Y/Y/N
6	F	7.9	Y	Y	N	Y	BC	Y	Y	Y	Y	Y	Y	N	N	0	16.9	NA	NA	N	CPS, Rt	Normal	42	19	Y/Y/N
7	M	7.3	Y	N	Y	N	BC	Y	Y	Y	N	N	Y	N	N	0	23.9	NA	NA	N	Normal	Normal	47	39	Y/Y/N
8	F	7.3	Y	Y	N	Y	BC	Y	N	Y	N	Y	N	Y	N	0	20.1	Neg	0.796	N	AS	Abnormal (Bil. frontal and parietal regions)	44	90	Y/Y/N
9	M	15.8	N	N	N	N	Sz	Y	Y	N	Y	N	N	N	Y	0	30.6	NA	0.647	N	BS/CPS, Rt	Abnormal (Rt thalamus, putamen, frontal, and temporal regions)	55	15	Y/Y/Y
10	F	17.5	Y	N	N	Y	BC	Y	N	N	N	Y	Y	Y	N	10	49.3	NA	0.644	N	Normal	Normal	68	7	Y/Y/Y
11	F	17.9	N	N	N	N	BC	Y	Y	Y	Y	Y	N	Y	N	0	22.7	Pos	0.868	N	Normal	Normal	63	60	Y/Y/Y

A, autonomic disturbance; AS, absence of sleep spindles; BC, behavioral changes, including mood, and/or personality changes; Bil., bilateral, BS, background slowing; C, consciousness disturbance; CPS, continuous polymorphic slowing; CSF, cerebrospinal fluid; D, dystonic posture; F, female; G, gastrointestinal symptoms, including vomiting and/or diarrhea; H, headache; Hal, hallucinations, including visual and/or auditory; Hosp, hospitalization; IVIG, intravenous immunoglobulin; Lt, left; M, male; MP, methylprednisolone; N, no; NA, not available; Neg, negative; O, orofacial dyskinesia; OCB, oligoclonal band; Pos, positive; PP, plasmapheresis; Pt, patient; Rt, right; S, speechless; s/s, symptoms and signs; Sz, seizure; U, upper respiratory tract infection; WBC, white blood cell; Y, Yes. * Patient 4 was hospitalized at another hospital at disease onset and referred to our hospital later. ** The parents of patient 5 asked to transfer to another hospital for a second opinion.

**Table 2:** Summary of demographic data, clinical manifestations, treatment methods, neuroimaging findings, and clinical outcomes in 11 patients with anti-NMDA receptor encephalitis.

Anti-NMDA Receptor Encephalitis	Total Number, N = 11(%)
Gender, male: female	3:8
Age at disease onset, median (range)	7.9 years (2.5–17.9)
Prodromal symptoms and signs	
Fever, *n* (%)	5 (45.5)
Upper respiratory tract infection, *n* (%)	4 (36.4)
Gastrointestinal symptoms (vomiting and/or diarrhea), *n* (%)	4 (36.4)
Headache, *n* (%)	4 (36.4)
Initial symptoms and signs	
Seizures, *n* (%)	3 (27.3)
Behavioral changes (mood, and/or personality changes), *n* (%)	8 (72.7)
Overall clinical manifestations	
Behavioral changes (mood, and/or personality changes), *n* (%)	11 (100)
Speechless, *n* (%)	7 (63.6)
Orofacial dyskinesia, *n* (%)	7 (63.6)
Seizures, *n* (%)	6 (54.5)
Autonomic disturbance, *n* (%)	6 (54.5)
Dystonic posture, *n* (%)	6 (54.5)
Hallucination (visual and/or auditory), *n* (%)	4 (36.4)
Consciousness disturbance, *n* (%)	4 (36.4)
Hospitalization duration, median (range) *	55 days (26–73)
Initial EEG	
Normal, *n* (%)	4 (36.4)
Abnormal, *n* (%)	7 (63.6)
Initial brain MRI	
Normal, *n* (%)	7 (63.6)
Abnormal, *n* (%)	4 (36.4)
Time to treatment, median (range)	21 days (7–90)
Treatment	
Methylprednisolone pulse therapy, *n* (%)	11 (100)
Intravenous immunoglobulin (IVIG), *n* (%)	10 (90.9)
Plasmapheresis, *n* (%)	4 (36.4)
Tumor removal, *n* (%)	1 (9)
	
Long-term follow-up	Total number, N = 10
Follow-up period, median (range) Persistent seizures, *n* (%)	3.5 years (1–6) 3 (30)
Follow-up EEG at 6–12 months	
Normal, *n* (%)	3 (30)
Abnormal, *n* (%)	5 (50)
Not available, *n* (%)	2 (20)
NEOS score, median (range)	2 points (1–4)
mRS at last follow-up	
≤2	9 (90)
>3	1 (10)
CASE score at last follow-up	
≤5	10 (100%)
>5	0 (0%)

* We excluded 2 patients because they had been transferred to another hospital.

**Table 3:** Clinical outcomes for NEOS score at disease onset; mRS/CASE score at disease onset, 6 months, 12 months, and last follow-up evaluation of 11 patients with anti-NMDA receptor encephalitis.

Pt	Age at Disease Onset (Years)	Disease Onset (NEOS Score)	Disease Onset (mRS/CASE)	6 Months (mRS/CASE)	12 Months (mRS/CASE)	Last Follow-Up (mRS/CASE)	Follow-Up (Years)	Sequelae
1	7.1	2	3/7	3/7	2/2	0/0	3	No
2	3.2	4	3/6	4/7	3/5	3/4	6	ID, speech problem, Sz
3	2.5	2	3/6	1/1	1/1	0/0	6	No
4	13.8	NA *	2/3	1/1	0/0	0/0	5.5	No
5	15.5	NA **	3/7	NA	NA	NA	NA	NA
6	7.9	1	3/6	1/1	1/1	1/1	5.7	ADHD, mood disorder
7	7.3	1	2/2	0/0	0/0	0/0	1	No
8	7.3	4	2/3	1/1	0/0	0/0	3.3	No
9	15.8	2	4/7	1/3	1/2	1/1	3.6	Clumsy, Sz ***
10	17.5	2	3/5	0/0	0/0	0/0	1	No
11	17.9	3	3/8	1/2	1/2	1/2	1.9	Memory impaired

ADHD, attention deficit hyperactivity disorder; CASE, Clinical Assessment Scale in Autoimmune Encephalitis; ID, intellectual disability; mRS, modified Rankin Scale; NA, not available; NEOS, Anti-NMDA Receptor Encephalitis One-Year Functional Status; Pt, patient; Sz, seizure. * Patient 4 was hospitalized at another hospital at disease onset and referred to our hospital later. ** Patient 5 was hospitalized, remaining in the intensive care unit for 5 days, and the patient’s parents asked to transfer to another hospital. *** Patient 9 had no seizures under oxcarbazepine and clobazam after 32 months from disease onset.
